# Integrating genomic mutations and tumor-infiltrating lymphocytes improves prediction of response to trastuzumab-based adjuvant therapy in patients with HER2-positive breast cancer

**DOI:** 10.20517/cdr.2025.133

**Published:** 2025-09-08

**Authors:** Shuangshuang Lu, Yuliang Zhang, Yiwei Tong, Lan Shu, Renhong Huang, Yijin Gu, Chaofu Wang, Jianfeng Li, Kunwei Shen, Lei Dong, Xiaosong Chen

**Affiliations:** ^1^Department of General Surgery, Comprehensive Breast Health Center, Ruijin Hospital, Shanghai Jiao Tong University School of Medicine, Shanghai 200025, China.; ^2^Shanghai Institute of Hematology, State Key Laboratory of Medical Genomics, National Research Center for Translational Medicine at Shanghai, Ruijin Hospital, Shanghai Jiao Tong University School of Medicine, Shanghai 200025, China.; ^3^Department of Pathology, Ruijin Hospital, Shanghai Jiao Tong University School of Medicine, Shanghai 200025, China.; ^#^Authors contributed equally.

**Keywords:** Breast neoplasms, genomic mutation, tumor-infiltrating lymphocytes, survival, models

## Abstract

**Aim:** Resistance to trastuzumab remains a major barrier to cure in early-stage HER2-positive breast cancer (HER2+ BC). We investigated the impact of genomic alterations and tumor-infiltrating lymphocyte (TIL) density on treatment resistance and survival outcomes.

**Methods:** We retrospectively analyzed 315 patients with HER2+ BC who received adjuvant trastuzumab at Ruijin Hospital (2009-2019). Whole-exome sequencing and TIL scoring were performed on surgical specimens, and clinical and pathological data were collected. The Cancer Genome Atlas (TCGA) cohort was used for external validation. Genomic alterations and TIL density were compared between trastuzumab-sensitive and -resistant tumors. Survival analyses were conducted to identify prognostic biomarkers.

**Results:** After a median follow-up of 109.3 months, 67 tumors (21.3%) were trastuzumab-resistant, exhibiting lower TIL density (mean 19.8% *vs.* 26.3%, *P* = 0.001), higher mutation frequencies in *FLG*, *MAP1A*, *BRCA1*, *PTPRD*, *PAPPA2*, *NCOR2*, *FBXW7*, *MYH7*, and *VCAN*, and more frequent alterations in the TP53/NOTCH pathways compared with sensitive tumors (all *P* < 0.05). A 15-gene trastuzumab response-associated gene (TRAG) signature independently predicted poorer disease-free survival (DFS) in both our cohort (HR, 3.57, *P* < 0.001) and the TCGA cohort (HR, 4.99, *P* = 0.037). A high copy number alteration burden was associated with worse overall survival (HR, 2.49, *P* = 0.043), whereas TIL density > 10% was associated with improved DFS (HR, 2.44, *P* = 0.003). A prognostic model integrating tumor size, nodal status, estrogen receptor status, TILs, and the TRAG signature showed strong discriminatory power (c-index 0.743 in the training set; 0.915 in the validation set).

**Conclusion:** Genomic alterations and reduced TIL density underpin trastuzumab resistance. The novel TRAG signature and integrated prognostic model enhance risk stratification and may guide personalized adjuvant therapy in early-stage HER2+ BC.

## INTRODUCTION

Breast cancer (BC) remains the leading cause of cancer-related deaths among women worldwide^[1]^. Approximately 15%-20% of BCs exhibit amplification of the human epidermal growth factor receptor 2 (HER2), a molecular subtype historically associated with aggressive biology and poor survival^[2,3]^. The advent of trastuzumab - an anti-HER2 monoclonal antibody - has remarkably improved outcomes for HER2-positive breast cancer (HER2+ BC) patients. However, 20%-30% of these patients ultimately develop resistance to trastuzumab, leading to tumor recurrence or death^[4]^. Overcoming trastuzumab resistance is therefore critical to improving cure rates in HER2+ BC.

The mechanisms underlying trastuzumab resistance remain incompletely delineated. Preclinical evidence suggests that genomic alterations, including mutations within the HER receptor family and *PIK3CA*, may contribute to resistance^[5-11]^. A pooled analysis of 967 patients from five trials showed that *PIK3CA* mutations were associated with lower treatment response rates in HER2+ BC^[12]^. Other studies have indicated that an elevated HER2 copy number alteration (CNA) burden in tumor cells correlates with improved outcomes following anti-HER2 therapy^[13]^. Additionally, the tumor immune microenvironment plays a critical role in trastuzumab efficacy. Trastuzumab exerts therapeutic effects both by inhibiting HER2 signaling and by triggering immune responses, primarily through antibody-dependent cellular cytotoxicity (ADCC)^[14]^. Tumor-infiltrating lymphocytes (TILs) are mononuclear immune cells infiltrating the tumor microenvironment and are the primary components of tumor-immune interactions. Higher TIL density has been associated with improved prognosis in HER2+ early breast cancer (EBC)^[15]^.

Despite these advances, no genomic or immune biomarker has been adopted in routine clinical practice to reliably distinguish trastuzumab-resistant from trastuzumab-sensitive patients. This is due to limitations such as small study populations, inconsistent findings, lack of robust validation, and the absence of consensus on clinical utility.

To address these gaps, we performed whole-exome sequencing (WES) and standardized TIL assessment on surgical specimens from 315 HER2+ EBC patients who received adjuvant trastuzumab therapy following surgery. By systematically comparing genomic mutation profiles, pathway alterations, CNA burden, and TIL density between trastuzumab-sensitive and -resistant tumors, we aimed to identify potential drivers of resistance and establish a prognostic framework to guide individualized adjuvant treatment strategies. Findings were further validated using The Cancer Genome Atlas (TCGA) cohort.

## METHODS

We adhered to the TRIPOD Statement for transparent reporting of multivariable prediction model development and validation in individual prognosis or diagnosis^[16]^.

### Study population

We retrospectively included patients with invasive BC who underwent surgery between January 2009 and October 2019 at the Comprehensive Breast Health Center, Ruijin Hospital, Shanghai Jiao Tong University School of Medicine (RJBC). Eligible criteria: Patients were eligible if they had (1) a diagnosis of invasive HER2+ BC; (2) TNM stage I, II, or III disease; (3) completed one year of adjuvant trastuzumab therapy; (4) available breast tumor specimens for WES analysis; and (5) complete clinicopathological and follow-up data. Exclusion criteria: Patients were excluded if they had received neoadjuvant therapy, had not received trastuzumab adjuvant therapy, or were diagnosed with metastatic (stage IV) disease. Clinicopathological and follow-up data were obtained from the Shanghai Jiao Tong University Breast Cancer Database (SJTU-BCDB). Patients were stratified by year of surgery into training and validation cohorts at an approximate ratio of 8:2. The study was approved by the Ethical Committee of Ruijin Hospital, Shanghai Jiao Tong University School of Medicine. All procedures were conducted in accordance with the Declaration of Helsinki (1964) and its subsequent amendments.

### Pathological evaluations

Histopathology, immunohistochemistry (IHC), and fluorescence *in situ* hybridization (FISH) evaluations were performed by pathologists. Estrogen receptor (ER), progesterone receptor (PR), HER2, and Ki-67 expression were assessed by IHC, as described in our previous studies^[17]^. HER2 status was independently confirmed by two pathologists to minimize observer variability. Equivocal HER2 IHC cases were further evaluated using dual-probe FISH with the PathVysion HER2 DNA kit (Vysis Inc., Downers Grove, IL, USA). According to the latest ASCO/CAP guidelines^[18,19]^, ER- or PR-positive status was defined as ≥ 1% tumor cells showing definite nuclear staining. HER2 positivity was defined as IHC 2+ with FISH positivity or IHC 3+. Stromal TILs were evaluated on the hematoxylin-eosin-stained slides by two pathologists and recorded as the percentage of stromal immune cell infiltration within the tumor boundaries^[20]^. TILs > 10% were classified as high-level, while TILs ≤ 10% were classified as low-level.

### WES and data analysis

#### WES

Archival FFPE blocks of primary tumors and adjacent normal breast tissues were obtained. Genomic DNA was extracted using the QIAmp DNA FFPE Tissue Kit (Qiagen) following the manufacturer’s instructions. DNA quantity was measured with NanoDrop (Thermo Fisher), and quality was evaluated via gel electrophoresis. Samples containing > 200 ng of genomic DNA were qualified for library preparation. Libraries were constructed following Illumina protocols. Briefly, genomic DNA was fragmented, purified, end-repaired, adenylated at the 3′ ends, ligated to indexed paired-end adaptors, purified, and amplified by polymerase chain reaction (PCR). Exome capture for tumors in the training cohort was performed using the Twist Human Core Exome Kit (Twist), covering 32 megabases (Mb) of the human genome coding regions (GRCh37). For the validation cohort, exome capture was performed using the SureSelect Human All Exon V6 Kit, targeting 60 Mb. Captured libraries were sequenced on the Novaseq 6000 platform (Illumina Inc.) for 2 × 100 paired-end reads using Cycle Sequencing v3 Reagents (Illumina). Only samples with > 80% of bases achieving a quality score of ≥ Q30 were included in the analysis. Target sequencing depth was set at 300× for tumor samples and 150× for normal samples.

#### Data processing

Raw BCL files were converted into FASTQ files using CASAVA 1.8.2. Reads were aligned to the human reference genome (hg19) with BWA to generate BAM files, which were further processed for duplicate marking, realignment, and recalibration using Picard and GATK.

Somatic mutations were called using Lofreq (version 2), GATK4 UnifiedGenotyper, GATK4 HaplotypeCaller, GATK4 Mutect2, and Strelka (version 2.9). Variants were annotated with vcf2maf using data from COSMIC, ClinVar, and ExAC. A panel of 25 pooled normal samples was used to filter germline mutations. Single-nucleotide variants (SNVs) and insertions/deletions (INDELs) were filtered at variant allele fractions (VAF) ≥ 0.02 and VAF ≥ 0.05, respectively. Mutations were included for downstream analysis if they met the following criteria: classified as “HIGH” or “MODERATE” impact by SnpEff; alteration frequency < 0.01 in the 1000 Genomes Project, ESP6500, and ExAC database; and located in cancer-related genes listed in the COSMIC Cancer Gene Census (CGC). CNAs were analyzed using PureCN and CNVkit (version 0.9.10) with default settings.

### Follow up

Patient follow-up was conducted annually by a specialized BC nurse. According to the latest standardized definitions for efficacy endpoints (STEEP) criteria^[21]^, disease-free survival (DFS) was defined as the time from surgery to the first event, including contralateral BC, localregional recurrence, a second non-breast primary cancer, distant metastasis, or death from any cause. Overall survival (OS) was defined as the time from surgery to death from any cause. Recurrence was defined as localregional recurrence, distant metastasis, or death during or after adjuvant therapy. Primary resistance was defined as recurrence (excluding brain metastasis) within one year after surgery, whereas non-primary resistance was defined as recurrence (excluding brain metastasis) occurring more than one year after surgery^[22]^.

### TCGA data

Publicly available genomic and clinical data for HER2+ BC cases were obtained from the TCGA database (https://www.cancer.gov/tcga). All data were de-identified and accessed in compliance with TCGA data-use policies. TCGA cases were included if they met the following criteria: (1) pathologically confirmed HER2+ BC; (2) received target therapy; and (3) had available WES data with corresponding clinical outcomes, including progression-free interval and OS.

### Statistical analysis

Categorical variables were analyzed using the chi-squared test (or Fisher’s exact test, where appropriate) and multinomial logistic regression. Continuous variables were analyzed with the Mann–Whitney U test. Survival outcomes were evaluated using Kaplan-Meier survival analysis and log-rank tests. Cox regression was used for univariate and multivariate survival analyses. The Akaike information criterion (AIC) was adopted to compare model fit. Prognostic performance was assessed using receiver operating characteristic (ROC) curves and the area under the curve (AUC). WES results were formatted into mutation annotation format (MAF) files and analyzed using the R package Maftools for variant classification, mutation frequency, cohort comparisons, genomic alteration oncoplots, lollipop plots of key genes, and oncogenic pathway analysis. Pathway enrichment analysis was performed with reference to the KEGG database. All statistical analyses were performed using SPSS version 24.0 (SPSS, Inc., Chicago, IL, USA), GraphPad Prism 9.0 (GraphPad Software, San Diego, CA, USA), and R version 4.0.5 (R Foundation for Statistical Computing, Vienna, Austria). Tests were two-tailed, and *P* < 0.05 was considered statistically significant.

## RESULTS

### Patient characteristics

**Figure 1:**
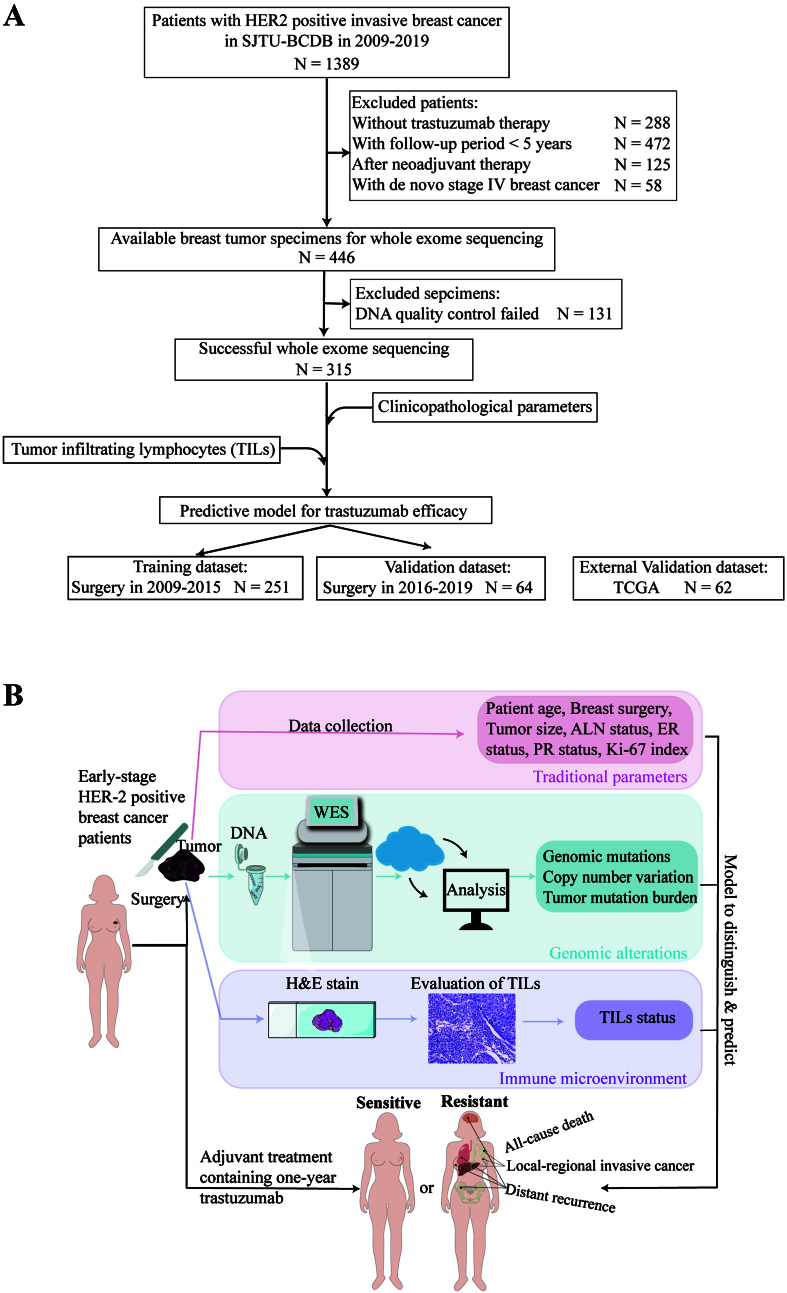
Flow diagram and study design. (A) Flow diagram; (B) Study design. HER2: Human epidermal growth factor receptor 2; SJTU-BCDC: Shanghai Jiao Tong University Breast Cancer Database; TCGA: The Cancer Genome Atlas; DNA: deoxyribonucleic acid; WES: whole-exome sequencing; TILs: tumor-infiltrating lymphocytes; H&E stain: hematoxylin and eosin stain; ALN: axillary lymph node; ER: estrogen receptor; PR: progesterone receptor.

Detailed patient enrollment is shown in Figure 1A, and the study design is illustrated in Figure 1B. Baseline characteristics of the enrolled patients are summarized in Table 1. The median age of the study population was 54 years (range, 27-91 years), and 239 patients (75.9%) underwent mastectomy. Tumors larger than 2.0 cm were present in 192 patients (61.0%), and 152 patients (48.3%) had axillary lymph node involvement. Histologic grade III tumors were observed in 209 patients (66.3%), and 172 patients (54.6%) were ER-negative. After a median follow-up of 109.3 months (range, 105.5-113.8 months), 248 patients (78.7%) remained recurrence-free and were classified as trastuzumab-sensitive, while 67 patients (21.3%) experienced recurrence and were deemed trastuzumab-resistant. Compared with sensitive cases, resistant tumors were more likely to be larger than 2.0 cm (80.6% *vs.* 55.6%, *P* < 0.001), ER-negative (73.1% *vs.* 49.6%, *P* < 0.001), and node-positive (59.7% *vs.* 45.2%, *P* = 0.048) [Table 1]. Among resistant cases, 61 were categorized as non-primary-resistant and 6 as primary-resistant [Supplementary Table 1].

**Table 1:** Demographical and clinical characteristics of HER2+ BC patients according to recurrent status in the training and validation cohorts

**Characteristics**	**Total** ***N* = 315 (%)**	**Sensitive cases** ***N* = 248 (%)**	**Resistant cases** ***N* = 67 (%)**	***P* value**
**Age (y/o)**				0. 560
≤ 50	115 (36.5)	88 (35.5)	27 (40.3)	
> 50	200 (63.5)	160 (64.5)	40 (59.7)	
**Breast surgery**				0.238
BCS	76 (24.1)	64 (25.8)	12 (17.9)	
Mastectomy	239 (75.9)	184 (74.2)	55 (82.1)	
**Tumor size (cm)**				< 0.001^*^
≤ 2.0	123 (39.0)	110 (44.4)	13 (19.4)	
> 2.0	192 (61.0)	138 (55.6)	54 (80.6)	
**ALN status**				0.048^*^
Negative	163 (51.7)	136 (54.8)	27 (40.3)	
Positive	152 (48.3)	112 (45.2)	40 (59.7)	
**Grade**				0.149
I-II	106 (33.7)	78 (31.5)	28 (41.8)	
III	209 (66.3)	170 (68.5)	39 (58.2)	
**ER**				< 0.001^*^
Negative	172 (54.6)	123 (49.6)	49 (73.1)	
Positive	143 (45.4)	125 (50.4)	18 (26.9)	
**PR**				0.003^*^
Negative	213 (67.6)	157 (63.3)	56 (83.6)	
Positive	102 (32.4)	91 (36.7)	11 (16.4)	
**HER2**				1.000
IHC++/FISH+	55 (17.5)	43 (17.3)	12 (17.9)	
IHC+++	260 (82.5)	205 (82.7)	55 (82.1)	
**Ki-67 (%)**				0.371
≤ 30	133 (42.2)	101 (40.7)	32 (47.8)	
> 30	182 (57.8)	147 (59.3)	35 (52.2)	

HER2: Human epidermal growth factor receptor 2; y/o: years old; BCS: breast-conserving surgery; ALN: axillary lymph node; ER: estrogen receptor; PR: progesterone receptor. ^*^Two-sided *P* < 0.05.

The training cohort comprised 251 patients who underwent surgery between 2009 and 2015, while the validation cohort included 64 patients treated between 2016 and 2019 [Figure 1A]. Baseline characteristics were comparable between the two cohorts [Supplementary Table 2].

### Genomic landscape and differential alterations

**Figure 2:**
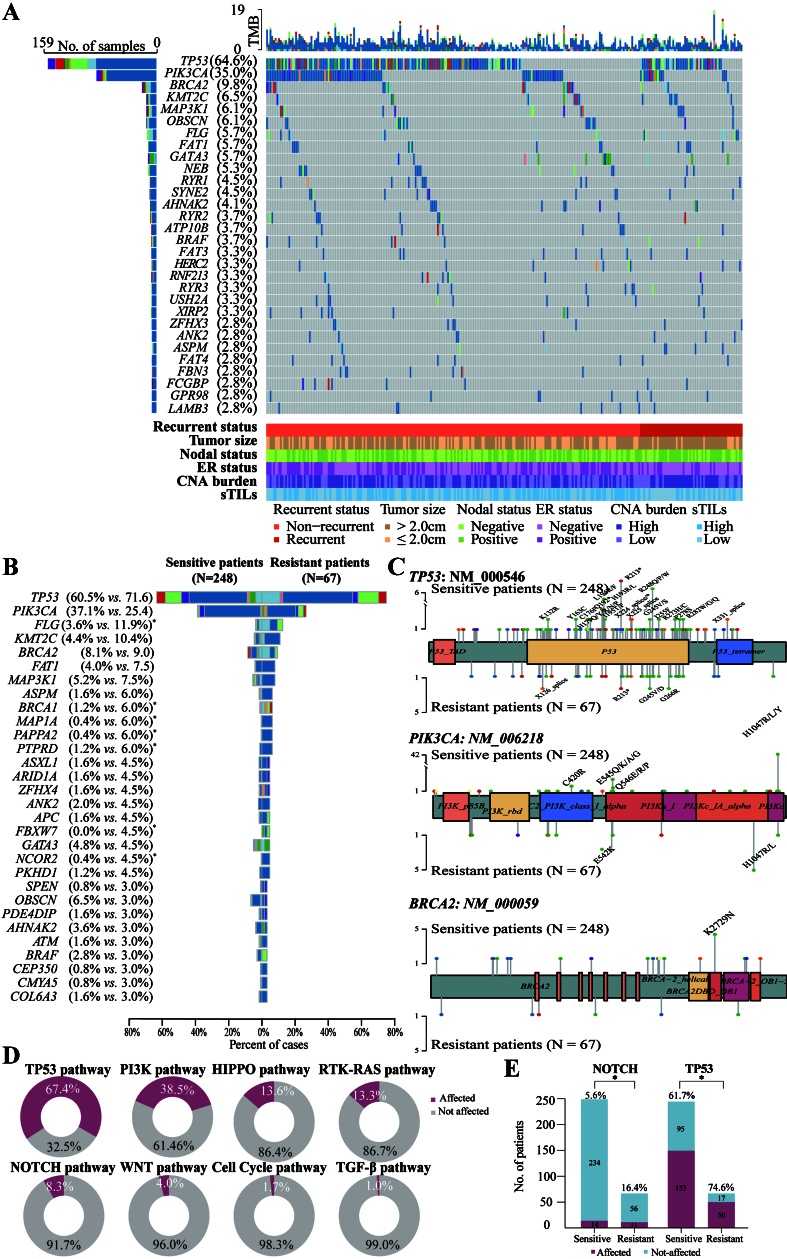
Mutational landscape of HER2-positive early-stage BC in the training cohort. (A) Oncoplot illustrating the 30 most frequently mutated genes in the whole population (*N* = 251), alongside relevant clinical features including recurrence status, tumor size, axillary lymph node involvement, ER status, CNA burden category, and TIL levels. Horizontal bar plot (top) shows the total number of mutations per tumor. Vertical bar plot (right) displays the number and proportion of patients with alterations per gene. Bar plot (bottom) shows prognosis, tumor size, lymph node status, ER status, TIL levels, and CNA burden category for each patient; (B) Comparison of mutation profiles between sensitive (non-recurrent, *N* = 248, left) and resistant (recurrent, *N* = 67, right) tumors. Genes are arranged in descending order of mutation frequency in the resistant group. Genes with significantly different mutation frequencies between the two groups are marked with an asterisk (^*^P < 0.05, Fisher’s exact test); (C) Lollipop plots showing positional enrichment of mutations within *TP53*, *PIK3CA*, and *BRCA2*, with differing hotspot variants in sensitive and resistant groups. Recurrent mutations are labeled; (D) Ring plots summarizing the frequency of disturbed oncogenic pathways. Gene lists for each pathway were determined by Sanchez-Vega and colleagues; (E) Resistant tumors showed significantly higher rates of alterations in the NOTCH (16.4% *vs.* 5.6%, *P* = 0.008) and TP53 (74.6% *vs.* 61.7%, *P* = 0.049) pathways compared with sensitive tumors. HER2: Human epidermal growth factor receptor 2; BC: breast cancer; ER: estrogen receptor; CNA: copy number alteration; TIL: tumor-infiltrating lymphocyte; sTILs: stromal tumor-infiltrating lymphocytes.

Comprehensive genomic profiling of 297 cancer-associated genes across the study population identified 1,051 missense, 120 nonsense, 128 frameshift, and 40 in-frame indel mutations [Supplementary Figure 1]. *TP53* mutations were the most prevalent cancer-related genomic alterations (64.6%), followed by nine genes with mutation frequencies ≥ 5%: *PIK3CA* (35.0%), *BRCA2* (9.8%), *KMT2C* (6.5%), *MAP3K1* (6.1%), *OBSCN* (6.1%), *FLG* (5.7%), *FAT1* (5.7%), *GATA3* (5.7%) and *NEB* (5.3%) [Figure 2A].

A comparison of mutational landscapes between resistant tumors (*n* = 67) and sensitive tumors (*n* = 248) is presented in Figure 2B. Several mutations occurred significantly more often in resistant tumors than in sensitive tumors, including *FLG* (11.9% *vs.* 3.6%, *P* = 0.013), *MAP1A* (6.0% *vs.* 0.4%, *P* = 0.008), *BRCA1* (6.0% *vs.* 1.2%, *P* = 0.039), *PTPRD* (6.0% *vs.* 1.2%, *P* = 0.039), *PAPPA2* (6.0% *vs.* 0.4%, *P* = 0.008), *NCOR2* (4.5% *vs.* 0.4%, *P* = 0.031), *FBXW7* (4.5% *vs.* 0.0%, *P* = 0.009), *MYH7* (3.0% *vs.* 0.0%, *P* = 0.045), and *VCAN* (3.0% *vs.* 0.0%, *P* = 0.045) [Figure 2B and Supplementary Table 3].

Positional enrichment analysis identified distinct recurrent variants. In *TP53*, sensitive tumors more frequently harbored nonsense mutations (p. Gln192^*^, p. Arg213^*^) and hotspot missense substitutions (p. Leu194Arg/Phe, p. Arg248Gln/Pro/Trp), whereas recurrent tumors carried a recurrent splice-site mutation (X126). Recurrent *PIK3CA* mutations (p. His1047Arg/Leu/Tyr) were observed in both cohorts. *BRCA2* mutations in sensitive tumors were limited to a single hotspot variant (p. Lys2729Asn) [Figure 2C].

Using the pathway classification proposed by Sanchez-Vega *et al.*, we observed frequent dysregulation of the TP53 pathway (67.4%), PI3K pathway (38.5%), and Hippo pathway (13.6%), followed by RTK-RAS (13.3%), NOTCH (8.3%), WNT (4.0%), Cell Cycle (1.7%), and TGF-β pathways (1.0%) [Figure 2D]^[23]^. Notably, alterations in the NOTCH pathway (16.4% *vs.* 5.6%, *P* = 0.008) and TP53 pathway (74.6% *vs.* 61.7%, *P* = 0.049) were significantly enriched in resistant tumors compared with sensitive tumors [Figure 2E and Supplementary Table 3].

CNA burden, defined as the proportion of the genome altered by CNA, had a median value of 38% (Q1-Q3: 21%-92%) in the training cohort. Based on this threshold, patients were categorized into CNA-high (> 38%, *n* = 157) and CNA-low (≤ 38%, *n* = 158) subgroups. High CNA burden significantly correlated with elevated Ki-67 (> 30%; *P* = 0.003) and histological grade 3 (*P* = 0.001), whereas other clinicopathological factors showed no correlation [Supplementary Table 4].

### Validation of genomic and pathway-level alterations in the TCGA cohort

We validated the identified gene- and pathway-level alterations in the TCGA-HER2+ cohort (*N* = 62, including 6 recurrent and 56 non-recurrent cases; baseline characteristics in Supplementary Table 5; mutational landscape in Supplementary Figure 2). However, the small sample size and low mutation rates substantially limited statistical power. The enrichment patterns observed in resistant tumors in our discovery cohort could not be independently confirmed in the TCGA dataset [Supplementary Table 3]. Similarly, pathway-level alterations, including those in the TP53 and NOTCH signaling axes, were not significantly replicated. These limitations underscore the strength of our large-scale cohort and highlight the need for future validation in multi-institutional datasets and prospective trials to confirm these findings and explore their mechanistic roles in trastuzumab resistance.

### Identification of trastuzumab response-associated genes

Lasso-based Cox regression analyses identified 15 genes (*FLG*, *MAP1A*, *ASPM*, *MEN1*, *PTEN*, *SMAD2*, *SCN7A*, *BRCA1*, *CTNNA1*, *FYN*, *HRNR*, *MRE11*, *RNF43*, *DCTN1*, and *PCLO*) whose mutations were significantly associated with reduced DFS in the training cohort (*P* < 0.05). Additionally, alterations in *VCAN*, *PRR16*, *SCN7A*, *ASTN1*, *BRWD1*, *SMG1*, *PTPRD*, and *FAM20C* correlated with poor OS (*P* < 0.05). These fifteen genes were consolidated into a trastuzumab response-associated gene (TRAG) signature. Tumors carrying mutations in at least one of the 15 genes were classified as TRAG-mutant, while those without such mutations were classified as TRAG–wild type. In the entire cohort, 271 tumors (86.0%) were TRAG wild-type, and 44 tumors (14.0%) were TRAG-mutant. There was no significant association between TRAG status and other clinicopathological features [Supplementary Table 6].

In the training cohort, patients with TRAG-wild-type tumors had 5- and 8-year OS rates of 92.5% (95%CI: 89.1%-96.1%) and 91.6% (95%CI: 87.9%-95.4%), and DFS rates of 87.4% (95%CI: 83.0%-91.9%) and 84.6% (95%CI: 79.9%-89.5%), respectively. In contrast, the TRAG-mutant group demonstrated substantially lower 5- and 8-year OS rates of 86.5% (95%CI: 76.1%-98.2%) and 83.8% (95%CI: 72.7%-96.5%), and DFS rates of 56.8% (95%CI: 42.8%-75.2%) and 54.1% (95%CI: 40.2%-72.8%), respectively.

**Figure 3:**
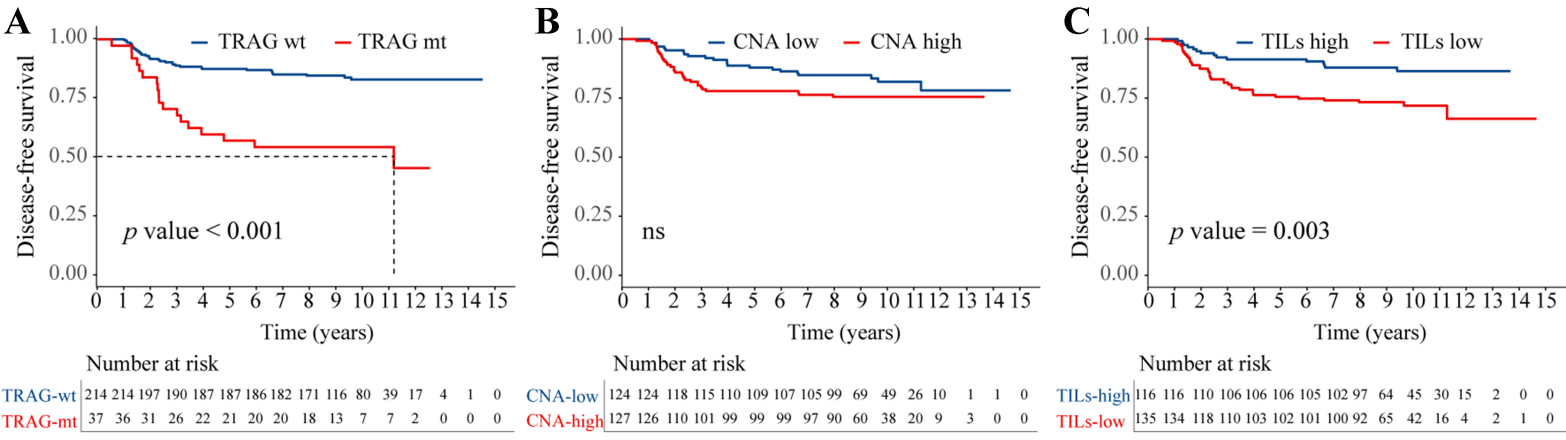
Association of TRAG signature status, CNA burden, and TIL levels with DFS in the training cohort. Kaplan-Meier estimates of DFS according to: (A) TRAG status (wild-type *vs.* mutant) (HR = 3.57; 95%CI: 1.58-8.02; *P* < 0.001); (B) CNA burden (low *vs.* high) (HR = 1.51; 95%CI: 0.88-2.59; *P* = 0.137); and (C) TIL level (low *vs.* high) (HR = 2.44; 95%CI: 1.34-4.45, *P* = 0.003). wt: Wild-type; mt: mutant; ns: not significant; HR: hazard ratio; DFS: disease-free survival; CI: confidence interval; CNA: copy number alteration; TILs: tumor-infiltrating lymphocytes; TRAG: trastuzumab response-associated gene; TRAG: We derived a 15-gene signature associated with trastuzumab response (*FLG*, *MAP1A*, *ASPM*, *MEN1*, *PTEN*, *SMAD2*, *SCN7A*, *BRCA1*, *CTNNA1*, *FYN*, *HRNR*, *MRE11*, *RNF43*, *DCTN1*, and *PCLO*).

TRAG status was significantly associated with DFS (HR = 3.57, 95%CI: 1.58-8.02, *P* < 0.001) [Figure 3A], and this association remained consistent across major subgroups [Supplementary Figure 3]. Although CNA levels were not significantly associated with DFS [Figure 3B], CNA burden showed a significant association with OS (HR = 2.49, 95%CI: 1.03-6.00, *P* = 0.043).

### Association between TILs and treatment response

The median density of TILs in the RJBC cohort was 10% [interquartile range (IQR), 5%-40%]. Using this threshold, 150 patients (47.6%) were classified as having high TILs (TILs > 10%) and 165 patients (52.4%) as having low TILs (TILs ≤ 10%). No significant differences were observed between the low- and high- TIL groups in terms of age, tumor size, node status, or ER status [Supplementary Table 7].

When comparing trastuzumab-resistant and -sensitive tumors, the sensitive group exhibited significantly higher TIL density [mean (SD), 26.3% (24.1%) *vs.* 19.8% (25.7%), *P* = 0.001] [Supplementary Figure 4].

In the training cohort, high TILs were associated with improved survival, with 5-year DFS and OS rates of 91.4% and 94.0%, respectively, compared with 75.6% and 89.6% in the low-TIL group. Low TILs independently predicted worse DFS (HR = 2.44, 95%CI: 1.34-4.45, *P* = 0.003) [Figure 3C], while no significant difference in OS was observed (data not shown). Subgroup analyses revealed no significant interactions between TILs and other clinical variables [Supplementary Figure 5].

### Construction and validation of a novel prognostic model integrating TRAG, TILs and other clinicopathological parameters

**Figure 4:**
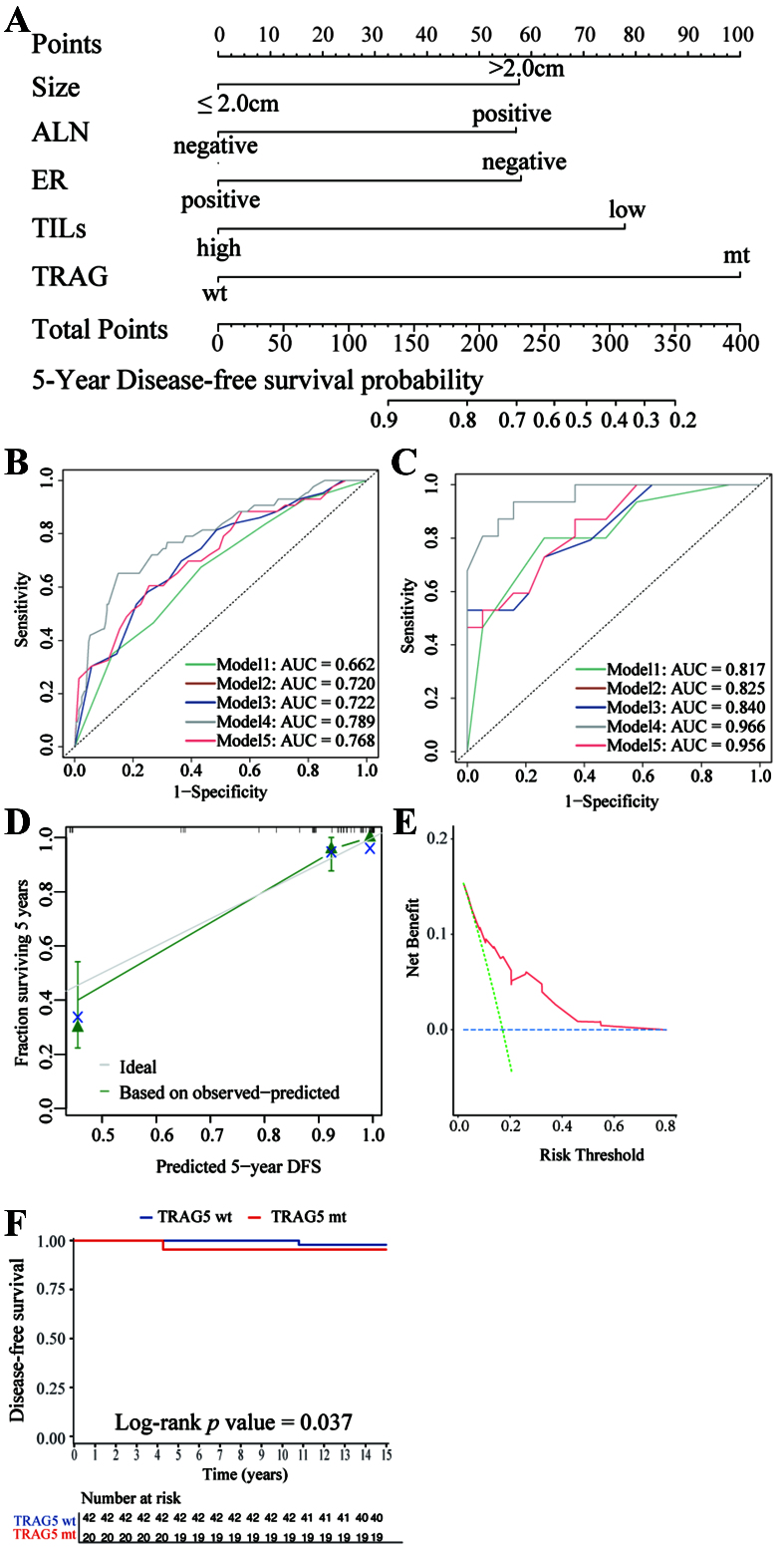
Visualization of the novel model and its performance. (A) Nomogram of the novel model for predicting 5-year DFS in HER2-positive early-stage BC patients receiving 1-year trastuzumab-based adjuvant treatment; (B) ROC curves in the training cohort for: Model 1 (tumor size, ALN status, ER status; green curve), Model 2 (Model 1 + TILs; brown curve), Model 3 (Model 2 + CNA level; blue curve), Model 4 (our novel 5-variable model: tumor size, ALN status, ER status, TILs, TRAG status; grey curve), and Model 5 (Model 3 + TRAG status; red curve). The performance of the novel model was evaluated by AUC; (C) ROC curves and AUC of Models 1-5 in the internal validation cohort; (D) Calibration curve in the validation cohort; (E) DCA in the validation cohort; (F) Kaplan-Meier estimates of DFS according to TRAG status (wild-type *vs.* mutant) in the TCGA cohort. ALN: Axillary lymph node; ER: estrogen receptor; TILs: tumor-infiltrating lymphocytes; TRAG: trastuzumab response-associated gene; DFS: disease-free survival; AUC: area under the receiver operating characteristic curve; wt: wild-type; mt: mutant; TCGA: The Cancer Genome Atlas; ROC: receiver operating characteristic curve; DCA: decision curve analysis.

Based on key findings from our cohort, we developed a novel prognostic model for 5-year DFS incorporating TIL level (≤ 10% *vs.* > 10%), TRAG status (mutant *vs.* wild-type), and clinicopathological features, including tumor size (> 2.0 cm *vs.* ≤ 2.0 cm), ALN status (positive *vs.* negative), and ER status (negative *vs.* positive) [Figure 4A and Supplementary Table 8]. This model achieved the lowest AIC (532.85) and the highest AUC (0.789) for predicting 5-year DFS compared with alternative models [Table 2 and Figure 4B]. Internal validation confirmed the model’s discriminative capacity, with a concordance index (C-statistic) of 0.915 (95%CI: 0.864-0.965) [Figure 4C]. Calibration plots demonstrated close agreement between predicted and observed 5-year DFS probabilities [Figure 4D]. Both integrated discrimination improvement (IDI) and continuous net reclassification improvement (NRI) analyses indicated that the novel 5-variable model outperformed simpler clinical or genomic models (data not shown). Decision curve analysis (DCA) further supported the model’s clinical utility by demonstrating a higher net benefit compared with strategies of treating all or no patients [Figure 4E].

**Table 2:** Comparison of prognostic models for DFS in our cohort using different combinations of CPs and molecular biomarkers

**Model, formula, and features**	**HR (95%CI)**	***P* value**	**AIC**	**LRT *P* value**
**Model 1: CP features**				
Tumor size (> 2.0 cm *vs.* ≤ 2.0 cm)	2.51 (1.31-4.80)	0.005	553.42	< 0.001^**^
ALN status (positive *vs.* negative)	1.67 (0.96-2.93)	0.071		
ER (negative *vs.* positive)	2.24 (1.26-4.00)	0.006		
**Model 2: CP features + TILs**				
Tumor size (> 2.0 cm *vs.* ≤ 2.0 cm)	2.51 (1.32-4.80)	0.005	545.28	< 0.001^**^
ALN status (positive *vs.* negative)	1.77 (1.01-3.11)	0.045		
ER (negative *vs.* positive)	2.15 (1.21-3.84)	0.009		
TILs (low *vs.* high)	2.53 (1.39-4.61)	0.002		
**Model 3: CP features + TILs + CNA**				
Tumor size (> 2.0 cm *vs.* ≤ 2.0 cm)	2.51 (1.31-4.78)	0.005	545.73	< 0.001^**^
ALN status (positive *vs.* negative)	1.87 (1.06-3.30)	0.030		
ER (negative *vs.* positive)	2.15 (1.20-3.83)	0.010		
TILs (low *vs.* high)	2.44 (1.33-4.46)	0.004		
CNA (high *vs.* low)	1.42 (0.81-2.48)	0.216		
**Model 4: CP features + TILs + TRAG (our novel model)**				
Tumor size (> 2.0 cm *vs.* ≤ 2.0 cm)	2.04 (1.05-3.96)	0.034	532.85	1 [reference]
ALN status (positive *vs.* negative)	2.03 (1.15-3.58)	0.015		
ER (negative *vs.* positive)	2.05 (1.15-3.66)	0.015		
TILs (low *vs.* high)	2.63 (1.44-4.81)	0.002		
TRAG mutation (yes *vs.* no)	3.45 (1.92-6.23)	< 0.001		
**Model 5: CP features + TILs + CNA + TRAG**				
Tumor size (> 2.0 cm *vs.* ≤ 2.0 cm)	2.04 (1.05-3.96)	0.035	533.85	0.377
ALN status (positive *vs.* negative)	2.03 (1.15-3.57)	0.014		
ER (negative *vs.* positive)	2.03 (1.13-3.62)	0.017		
TILs (low *vs.* high)	2.57 (1.40-4.71)	0.002		
CNA (high *vs.* low)	1.28 (0.74-2.23)	0.380		
TRAG mutation (yes *vs.* no)	3.34 (1.85-6.05)	< 0.001		

DFS: Disease-free survival; CP: clinicopathologic parameter; HR: hazard Ratio; CI: confidence interval; AIC: Akaike information criterion; LRT: likelihood ratio test; ALN: axillary lymph node; ER: estrogen receptor; TILs: tumor-infiltrating lymphocytes; CNA: copy number alteration; TRAG: trastuzumab response-associated gene. TRAG: We derived a 15-gene signature associated with trastuzumab response (*FLG*, *MAP1A*, *ASPM*, *MEN1*, *PTEN*, *SMAD2*, *SCN7A*, *BRCA1*, *CTNNA1*, *FYN*, *HRNR*, *MRE11*, *RNF43*, *DCTN1*, and *PCLO*). ^**^Two-sided *P* < 0.001.

Due to the absence of TILs data in the TCGA cohort, external validation of the complete model could not be performed. Nonetheless, TRAG mutation status alone significantly stratified patients by DFS in the TCGA cohort (HR = 4.99; 95%CI: 1.20-20.75, *P* = 0.037), confirming its independent prognostic relevance [Figure 4F].

## DISCUSSION

This study provides novel insights into the genomic and immune mechanisms underlying trastuzumab resistance in HER2+ EBC. By integrating WES analysis and TIL quantification in a real-world cohort with long-term follow-up, we identified distinct genomic profiles between treatment-resistant and -sensitive tumors. Importantly, we identified a 15-gene TRAG signature and developed a composite prognostic model incorporating TRAG status, TIL levels, and clinicopathologic features. This integrative model outperformed conventional clinical indicators in prognostic accuracy and may serve as a practical tool for individualized risk stratification in HER2+ BC.

Whole-exome profiling revealed nine genes - *FLG*, *MAP1A*, *BRCA1*, *PTPRD*, *PAPPA2*, *NCOR2*, *FBXW7*, *MYH7*, and *VCAN* - enriched in resistant tumors*.* Mechanistically, *BRCA1* loss compromises homologous recombination, fostering heterogeneous resistant clones under therapeutic pressure^[24]^. Previous studies have also reported that *BRCA*-deficient HER2+ tumors exhibit more aggressive phenotypes and worse 5-year OS^[25]^. *PTPRD* inactivation may activate JAK/STAT and PI3K/AKT signaling^[26]^, thereby counteracting trastuzumab-induced apoptosis^[27,28]^. *PAPPA2*, a potential immune checkpoint predictor^[29]^, may influence trastuzumab efficacy by modulating the IGFBP/IGF axis^[30,31]^. *NCOR2* enables tumor cells to evade immune surveillance by suppressing MHC class I expression and impairing CD8+ T cell activity, thereby promoting metastasis; notably, high NCOR2 expression correlates with shortened metastasis-free survival in BC^[32]^. *FBXW7*, a tumor suppressor, counters trastuzumab resistance by targeting cyclin E and regulating mTOR and NOTCH signaling^[33-35]^. Although *FLG* and *VCAN* are less well explored in HER2+ BC, *FLG* mutations have been associated with higher tumor mutation load and immunogenicity in gastric cancer^[36]^, while *VCAN* alterations have been linked to prostate cancer progression and docetaxel resistance^[37,38]^. Their enrichment in resistant tumors warrants further mechanistic investigation.

At the pathway level, resistant tumors exhibited significant enrichment of NOTCH and TP53 pathway alterations. Preclinical studies demonstrated HER2-NOTCH crosstalk, in which HER2 inhibition activated Notch1, driving trastuzumab resistance^[39,40]^. *TP53,* a central genomic guardian, is frequently mutated (~70%) in HER2+ BC^[41]^. TP53 loss can activate PI3K/AKT pathways, promoting trastuzumab resistance and aggressive clinical behavior^[42]^. Collectively, these alterations generate a mutational landscape primed for therapeutic escape.

TILs, reflecting antitumor immune activity, were significantly lower in resistant tumors. Higher TIL levels (> 10%) were associated with improved DFS, supporting the notion that immunologically “hot” tumors are more responsive to trastuzumab, especially through ADCC^[14,43-45]^. These results emphasize that resistance is not solely driven by genomic alterations but is also shaped by the tumor immune microenvironment, highlighting a rationale for combining HER2-targeted therapies with immunomodulatory strategies.

To translate genomic complexity into clinical utility, we derived a 15-gene TRAG signature that independently predicted inferior DFS across both our cohort and the TCGA cohort. This signature comprises genes involved in chromatin remodeling, DNA damage repair, and immune regulation. Incorporating TRAG, TILs, tumor size, nodal status, and ER status produced a five-variable model with a validation c-index exceeding 0.9, outperforming traditional nomograms in terms of discrimination, calibration, and DCA.

Our study represents one of the largest WES-integrated cohorts of HER2+ EBC with long-term outcome data. Nevertheless, several limitations must be considered. First, although both internal and external validations were performed, the retrospective, single-center design and the limited size of the validation cohorts may introduce selection bias and constrain the generalizability of our findings. The retrospective nature also precludes causal inference, and the observed associations should be considered exploratory, requiring confirmation in prospective, multi-institutional studies. Second, functional validation of the 15-gene TRAG signature remains pending, though ongoing efforts using CRISPR-based gene editing and patient-derived organoids aim to elucidate the mechanistic roles of these genes in trastuzumab resistance. Third, while TIL assessment followed International TILs Working Group guidelines^[20]^, the lack of subpopulation resolution hinders a comprehensive understanding of immune dynamics in trastuzumab resistance. TILs comprise heterogeneous subsets, including tumor-suppressing cells (e.g., CD8+ T cells, NK cells) and tumor-promoting cells (e.g., Treg cells). In BC, CD8+ T cells are associated with enhanced therapeutic responses^[46]^ and survival^[47]^, whereas CD4+ regulatory T cells exert more complex effects, being linked to both favorable and unfavorable outcomes^[48-51]^. Future investigations should therefore prioritize the functional dissection of distinct TIL subsets. Fourth, external validation of the integrated genomic-immune-clinical model was restricted by the absence of TIL data in publicly available cohorts, underscoring the need for collaborative efforts to confirm its robustness across diverse populations. Finally, although our median follow-up exceeded nine years, late recurrences beyond this window cannot be excluded, highlighting the need for extended surveillance in future studies. It should also be noted that the present cohort comprised patients treated between 2009 and 2019, while next-generation anti-HER2 agents - including pertuzumab, TKIs, and nanosystem-based therapeutics^[52]^ - are now in routine use or under active investigation. The prognostic utility of our model in patients receiving these newer agents, therefore, requires dedicated validation.

In conclusion, we identified a novel TRAG signature associated with trastuzumab resistance and recurrence in HER2-positive early-stage BC. Our integrated prognostic model, combining genomic, immune, and clinical variables, demonstrated high predictive accuracy and may help guide individualized therapeutic strategies. Prospective validation and mechanistic studies are needed to refine its clinical applicability and further elucidate underlying biological pathways.

## DECLARATIONS

### Availability of data and materials

The data used and/or analyzed during the current study are available from the corresponding author upon reasonable request.

### Copyright

© The Author(s) 2025.
