# Supplementary material for: Integrating genomic mutations and tumor-infiltrating lymphocytes improves prediction of response to trastuzumab-based adjuvant therapy in patients with HER2-positive breast cancer
Source: Cancer Drug Resist. 2025 Sep 8;8:47. doi: 10.20517/cdr.2025.133 (PMC12462396; doi:10.20517/cdr.2025.133)
Supplement: Supplementary file 1 [file cdr-8-47-SupplementaryMaterials.pdf]

Supplementary Materials

Integrating genomic mutations and tumor-infiltrating lymphocytes improves prediction of response to trastuzumab-based adjuvant therapy in patients with HER2-positive breast cancer

Shuangshuang Lu[1,#], Yuliang Zhang[2,#], Yiwei Tong[1,#], Lan Shu[1,#], Renhong Huang[1,#], Yijin Gu[3], Chaofu Wang[3], Jianfeng Li[2], Kunwei Shen[1], Lei Dong[3], Xiaosong Chen[1]

[1]Department of General Surgery, Comprehensive Breast Health Center, Ruijin Hospital, Shanghai Jiao Tong University School of Medicine, Shanghai 200025, China.
[2]Shanghai Institute of Hematology, State Key Laboratory of Medical Genomics, National Research Center for Translational Medicine at Shanghai, Ruijin Hospital, Shanghai Jiao Tong University School of Medicine, Shanghai 200025, China.
[3]Department of Pathology, Ruijin Hospital, Shanghai Jiao Tong University School of Medicine, Shanghai 200025, China.
[#]Authors contributed equally.

Correspondence to: Dr. Kunwei Shen, Dr. Xiaosong Chen, Department of General Surgery, Comprehensive Breast Health Center, Ruijin Hospital, Shanghai Jiao Tong University School of Medicine, Shanghai 200025, China. E-mail: kwshen@medmail.com.cn; chenxiaosong0156@hotmail.com; Dr. Lei Dong, Department of Pathology, Ruijin Hospital, Shanghai Jiao Tong University School of Medicine, Shanghai 200025, China. E-mail: dl11968@rjh.com.cn

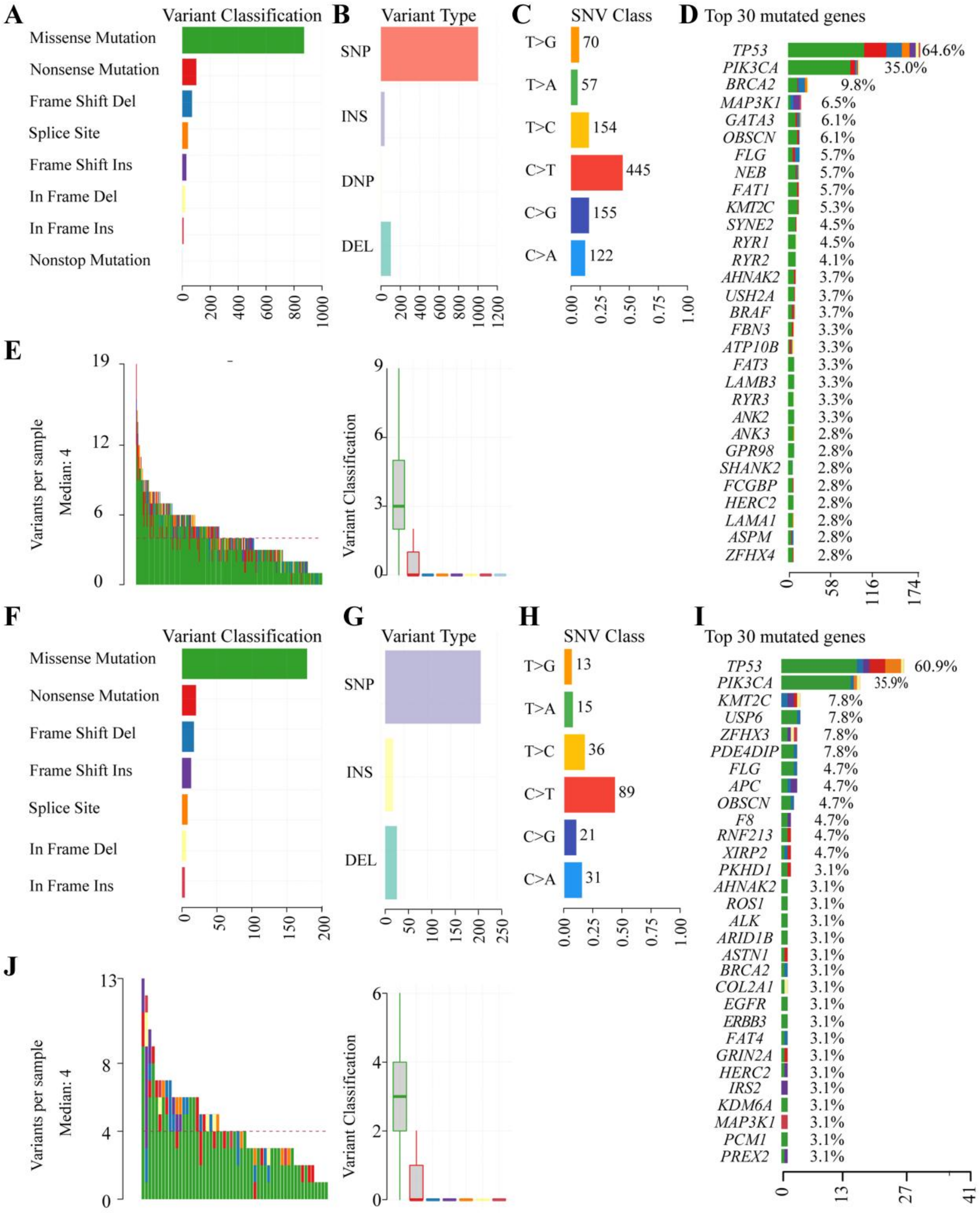

Supplementary Figure 1. Summary of the variants from the following aspects in the training cohort (A-E) and the validation cohort (F-J): variant classification (A and F), variant type (B and G), SNV class (C and H), the top 30 mutated genes (D and I), and variants per sample (E and J).

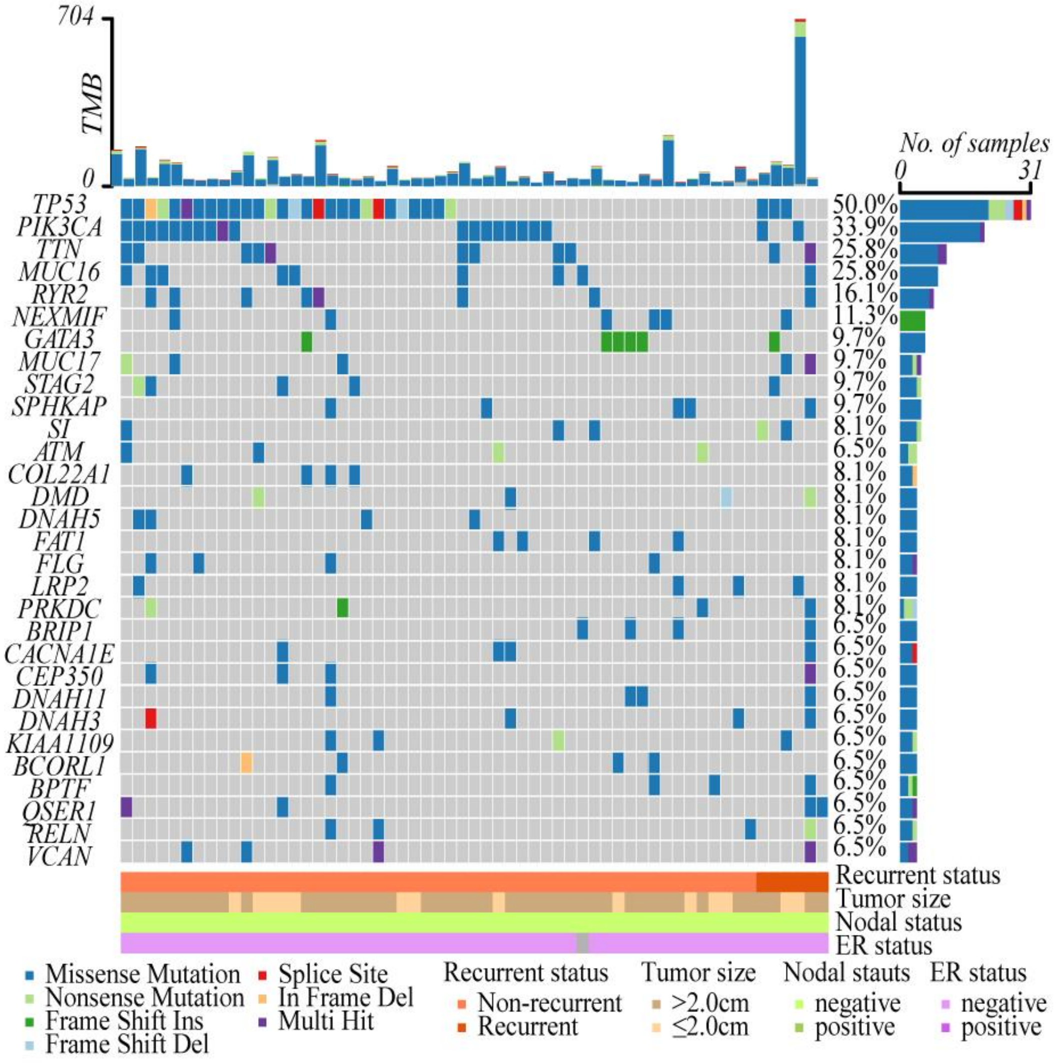

Supplementary Figure 2. Mutational landscape of early-stage HER2 positive breast cancer in the TCGA cohort. Oncoplot displays the mutational status and classification of the top 30 frequently mutated genes accompanied by their frequency in the TCGA cohort ($N = 62$). TCGA: The Cancer Genome Atlas.

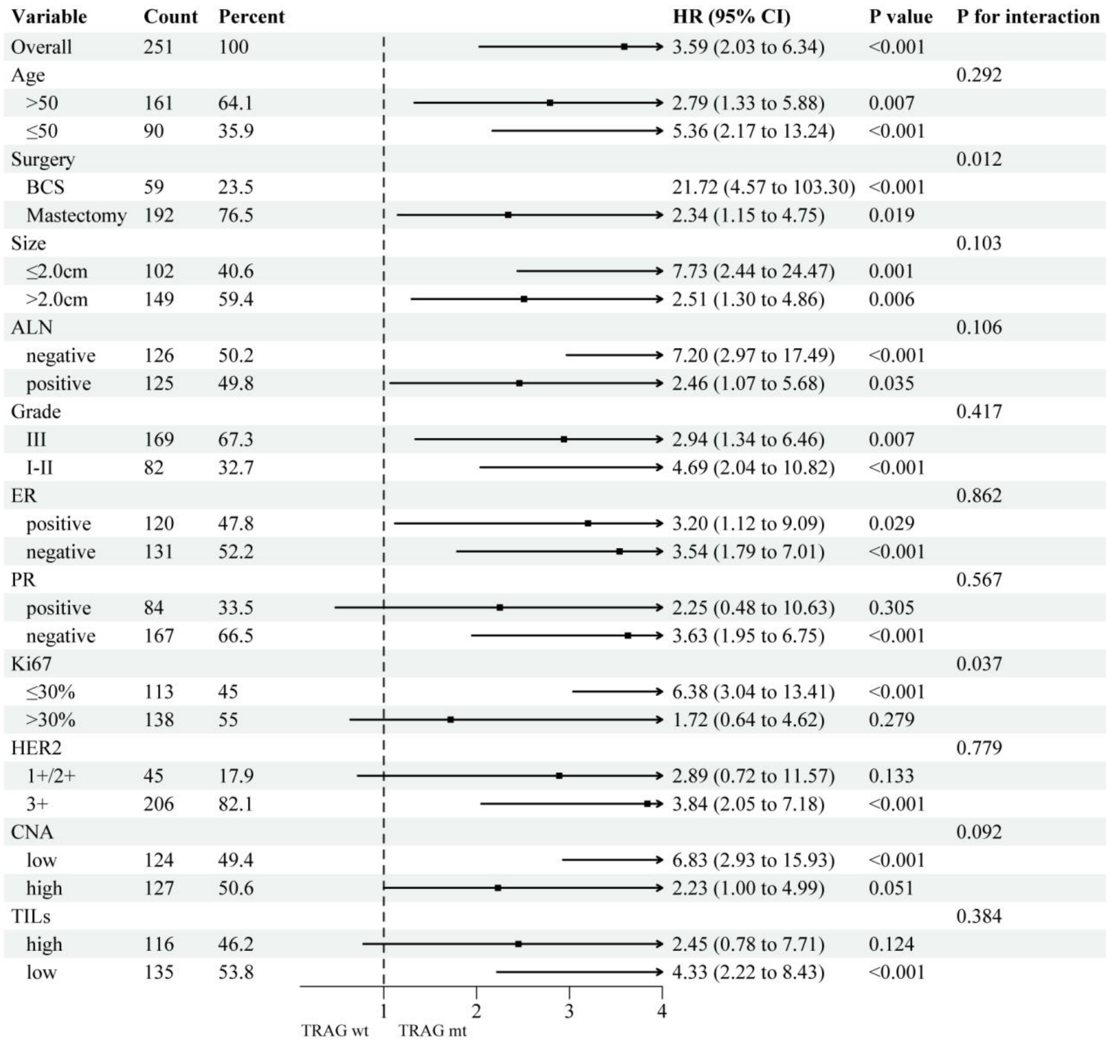

Supplementary Figure 3. Subgroup analysis of the prognostic value of trastuzumab response-associated gene signature (TRAG) status in the training cohort. Forest plot of the prognostic value of TRAG status for disease-free survival (DFS) according to baseline characteristics in the training cohort. HR: Hazard ratio; CI: confidence interval; BCS: breast conserving surgery; ALN: axillary lymph node; ER: estrogen receptor; PR: progesterone receptor; HER2: human epidermal growth factor receptor 2; CNA: copy number alteration; TILs: tumor-infiltrating lymphocytes; TRAG: trastuzumab-response-associated gene signature; wt: wild-type; mt: mutant.

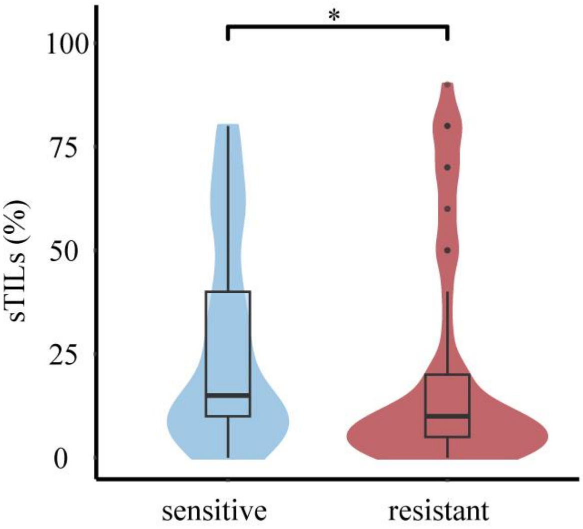

Supplementary Figure 4. Distribution of tumor infiltrating lymphocytes (TILs) in trastuzumab-sensitive tumors versus trastuzumab-resistant tumors. Violin plot showing the distribution of tumor-infiltrating lymphocytes (TILs) score in trastuzumab-sensitive tumors compared with trastuzumab-resistant tumors. The plot illustrates the median, interquartile range, and density distribution of TIL percentages in each group. *Two-sided $P < 0.05$.

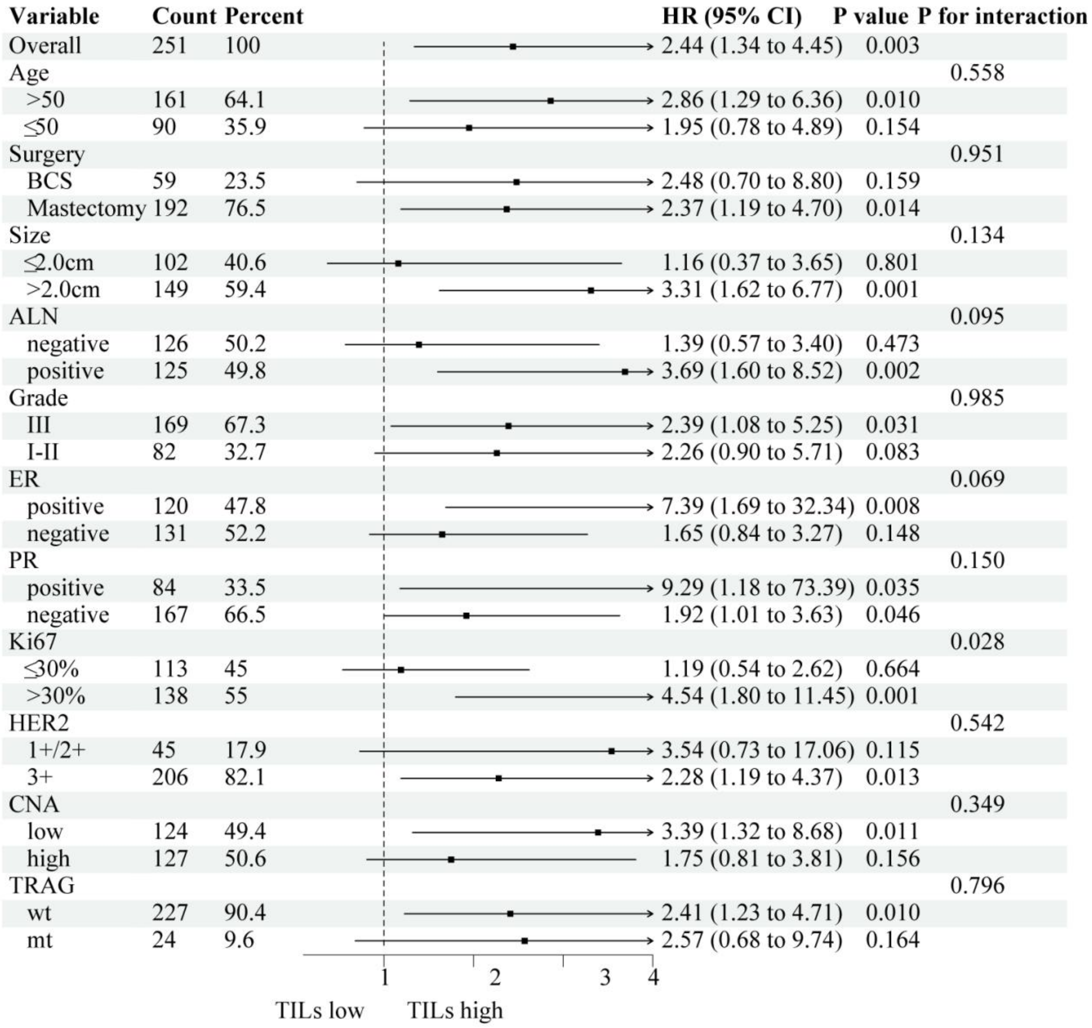

Supplementary Figure 5. Subgroup analysis of the prognostic value of tumor infiltrating lymphocytes (TILs) in the training cohort. Forest plot of the prognostic value of TILs for disease-free survival according to baseline characteristics in the training cohort. Patients were categorized into TILs-high subgroup (TILs >10%) and TILs-low subgroup (TILs ≤ 10%). HR: Hazard ratio; CI: confidence interval; BCS: breast conserving surgery; ALN: axillary lymph node; ER: estrogen receptor; PR: progesterone receptor; HER2: human epidermal growth factor receptor 2; CNA: copy number alteration; TRAG: trastuzumab-response-associated gene signature; wt: wild-type; mt: mutant; TILs: tumor-infiltrating lymphocytes.

Supplementary Table 1. Demographical characteristics of the HER2 positive breast cancer patients according to resistance classification

Characteristics	Total N = 67 (%)	Primary resistance N = 6 (%)	Non-primary resistance N = 61 (%)	P value
**Age (y/o)**				0. 069
≤ 50	27 (40.3)	5 (83.3)	22 (36.1)	
> 50	40 (59.7)	1 (16.7)	39 (63.9)	
**Breast surgery**				0.521
BCS	12 (17.9)	0 (0.0)	12 (19.7)	
Mastectomy	55 (82.1)	6 (100.0)	49 (80.3)	
**Tumor size (cm)**				0.472
≤ 2.0	13 (19.4)	0 (0.0)	13 (21.3)	
> 2.0	54 (80.6)	6 (100.0)	48 (78.7)	
**ALN status**				1.000
Negative	27 (40.3)	2 (33.3)	25 (41.0)	
Positive	40 (59.7)	4 (66.7)	36 (59.0)	
**Grade**				0.082
I-II	28 (41.8)	0 (0.0)	28 (45.9)	
III	39 (58.2)	6 (100.0)	33 (54.1)	
**ER**				0.283
Negative	49 (73.1)	6 (100.0)	43 (70.5)	
Positive	18 (26.9)	0 (0.0)	18 (29.5)	
**PR**				0.575
Negative	56 (83.6)	6 (100.0)	50 (82.0)	
Positive	11 (16.4)	0 (0.0)	11 (18.0)	
**HER2**				1.000
IHC++/FISH+	12 (17.9)	0 (0.0)	28 (45.9)	
IHC+++	55 (82.1)	6 (100.0)	33 (54.1)	
**Ki-67 (%)**				0.754
≤ 30	32 (47.8)	2 (33.3)	30 (49.2)	
> 30	35 (52.2)	4 (66.7)	31 (50.8)	

HER2: Human epidermal growth factor receptor 2; y/o: years old; BCS: breast conserving surgery; ALN: axillary lymph node; ER: estrogen receptor; PR: progesterone receptor.

Definitions: Primary resistance was defined as disease recurrence, excluding brain metastasis, within one year after surgery. Non-primary resistance was defined as recurrence, excluding brain metastasis, occurring more than one year after surgery.

Supplementary Table 2. Demographical characteristics of the HER2 positive breast cancer patients according to recurrent status in the training and validation cohorts

		Training (N = 251)			Validation (N = 64)		
**Characteristics**	**Total N = 315 (%)**	**Non-recurrent N = 198 (%)**	**Recurrent N = 53 (%)**	**P value**	**Non-recurrent N = 50 (%)**	**Recurrent N = 14 (%)**	**P value**
**Age (y/o)**				0. 873			0.523
≤ 50	115 (36.5)	70 (35.4)	20 (37.7)		18 (36.0)	7 (50.0)	
> 50	200 (63.5)	128 (64.6)	33 (62.3)		32 (64.0)	7 (50.0)	
**Breast surgery**				0.475			0.404
BCS	76 (24.1)	49 (24.7)	10 (18.9)		15 (30.0)	2 (14.3)	
Mastectomy	239 (75.9)	149 (75.3)	43 (81.1)		35 (70.0)	12 (85.7)	
**Tumor size (cm)**				0.004*			0.046*
≤ 2.0	123 (39.0)	90 (45.5)	12 (22.6)		20 (40.0)	1 (7.1)	
> 2.0	192 (61.0)	108 (54.5)	41 (77.4)		30 (60.0)	13 (92.9)	
**ALN status**				0.059			0.716
Negative	163 (51.7)	106 (53.5)	20 (37.7)		30 (60.0)	7 (50.0)	
Positive	152 (48.3)	92 (46.5)	33 (62.3)		20 (40.0)	7 (50.0)	
**Grade**				0.041*			0.639
I-II	106 (33.7)	58 (29.3)	24 (45.3)		20 (40.0)	4 (28.6)	
III	209 (66.3)	140 (70.7)	29 (54.7)		30 (60.0)	10 (71.4)	
**ER**				0.015*			0.026*
Negative	172 (54.6)	95 (48.0)	36 (67.9)		22 (44.0)	13 (92.9)	
Positive	143 (45.4)	103 (52.0)	17 (32.1)		28 (56.0)	1 (7.1)	
**PR**				0.018*			0.101
Negative	213 (67.6)	124 (62.6)	43 (81.1)		33 (66.0)	13 (92.9)	
Positive	102 (32.4)	74 (37.4)	10 (18.9)		17 (34.0)	1 (7.1)	
**HER2**				0.999			0.795
IHC++/FISH+	55 (17.5)	36 (18.2)	9 (17.0)		7 (14.0)	3 (21.4)	
IHC+++	260 (82.5)	162 (81.8)	44 (83.0)		43 (86.0)	11 (78.6)	
**Ki-67 (%)**				0.149			0.568
≤ 30	133 (42.2)	84 (42.4)	29 (54.7)		17 (34.0)	3 (21.4)	
> 30	182 (57.8)	114 (57.6)	24 (45.3)		33 (66.0)	11 (78.6)	

HER2: Human epidermal growth factor receptor 2; y/o: years old; BCS: breast conserving surgery; ALN: axillary lymph node; ER: estrogen receptor; PR: progesterone receptor. *Two-sided $P < 0.05$.

Supplementary Table 3. Differential gene mutations between sensitive tumors and resistant tumors

Alterations	RJBC cohort				TCGA cohort			
	All tumors N = 315 (%)	Sensitive tumors N = 248 (%)	Resistant tumors N = 67 (%)	P value	All tumors N = 62 (%)	Sensitive tumors N = 56 (%)	Resistant tumors N = 6 (%)	P value
**BRCA1**				0.039*				0.097
wild-type	308 (97.8)	245 (98.8)	63 (94.0)		61 (98.4)	56 (100.0)	5 (83.3)	
mutant	7 (2.2)	3 (1.2)	4 (6.0)		1 (1.6)	0 (0.0)	1 (16.7)	
**FBXW7**				0.009*				-
wild-type	313 (99.4)	248 (100.0)	64 (95.5)		-	-	-	
mutant	2 (0.6)	0 (0.0)	3 (4.5)		-	-	-	
**FLG**				0.013*				1.000
wild-type	298 (94.6)	239 (96.4)	59 (88.1)		57 (91.9)	51 (91.1)	6 (100.0)	
mutant	17 (5.4)	9 (3.6)	8 (11. 9)		5 (8.1)	5 (8.9)	0 (0.0)	
**MAP1A**				0.008*				1.000
wild-type	310 (98.4)	247 (99.6)	63 (94.0)		60 (96.8)	54 (96.4)	6 (100.0)	
mutant	5 (1.6)	1 (0.4)	4 (6.0)		2 (3.2)	2 (3.6)	0 (0.0)	
**MYH7**				0.045*				-
wild-type	313 (99.4)	248 (100.0)	65 (97.0)		-	-	-	
mutant	2 (0.6)	0 (0.0)	2 (3.0)		-	-	-	
**NCOR2**				0.031*				-
wild-type	311 (98.7)	247 (99.6)	64 (95.5)		-	-	-	
mutant	4 (1.3)	1 (0.4)	3 (4.5)		-	-	-	
**PAPPA2**				0.008*				0.267
wild-type	310 (98.4)	247 (99.6)	63 (94.0)		59 (95.2)	54 (96.4)	5 (83.3)	
mutant	5 (1.6)	1 (0.4)	4 (6.0)		3 (4.8)	2 (3.6)	1 (16.7)	
**PTPRD**				0.039*				-
wild-type	308 (97.8)	245 (98.8)	63 (94.0)		-	-	-	
mutant	7 (2.2)	3 (1.2)	4 (6.0)		-	-	-	
**VCAN**				0.045*				0.342
wild-type	313 (99.4)	248 (100.0)	65 (97.0)		58 (93.5)	53 (94.6)	5 (83.3)	
mutant	2 (0.6)	0 (0.0)	2 (3.0)		4 (6.5)	3 (5.4)	1 (16.7)	
**TP53 pathway**				0.049*				1.000
wild-type	112 (35.6)	95 (38.3)	17 (25.4)		29 (46.8)	26 (46.4)	3 (50.0)	
mutant	203 (64.4)	153 (61.7)	50 (74.6)		33 (53.2)	30 (53.6)	3 (50.0)	
**NOTCH pathway**				0.008*				0.328
wild-type	290 (92.1)	234 (94.4)	56 (83.6)		50 (80.6)	46 (82.1)	4 (66.7)	
mutant	25 (7.9)	14 (5.6)	11 (16.4)		12 (19.4)	10 (17.9)	2 (33.3)	
**TGF-β pathway**				1.000				0.043*
wild-type	312 (99.0)	246 (99.2)	66 (98.5)		58 (93.5)	54 (96.4)	4 (66.7)	
mutant	3 (1.0)	2 (0.8)	1 (1.5)		4 (6.5)	2 (3.6)	2 (33.3)	

RJBC: Ruijin Hospital Comprehensive Breast Health Center; TCGA: the Cancer Genome Atlas (TCGA). *Two-sided $P < 0.05$.

Supplementary Table 4. Baseline characteristics of RJBC cohort according to CNA level

Characteristics	Training (N = 251) Low-level N = 124 (%)	Training (N = 251) High-level N = 127 (%)	Training P value	Validation (N = 64) Low-level N = 34 (%)	Validation (N = 64) High-level N = 30 (%)	Validation P value
**Age (y/o)**			0.288			0.360
≤ 50	49 (39.5)	41 (32.3)		11 (32.4)	14 (46.7)	
> 50	75 (60.5)	86 (67.7)		23 (67.6)	16 (53.3)	
**Breast surgery**			0.093			0.763
BCS	23 (18.5)	36 (28.3)		8 (23.5)	9 (30.0)	
Mastectomy	101 (81.5)	91 (71.7)		26 (76.5)	21 (70.0)	
**Tumor size (cm)**			0.588			0.854
≤ 2.0	53 (42.7)	49 (38.6)		12 (35.3)	9 (30.0)	
> 2.0	71 (57.3)	78 (61.4)		22 (64.7)	21 (70.0)	
**ALN status**			0.850			0.937
Negative	61 (49.2)	65 (51.2)		19 (55.9)	18 (60.0)	
Positive	63 (50.8)	62 (48.8)		15 (44.1)	12 (40.0)	
**Grade**			0.001*			1.000
I-II	53 (42.7)	29 (22.8)		13 (38.2)	11 (36.7)	
III	71 (57.3)	98 (77.2)		21 (61.8)	19 (63.3)	
**ER**			0.416			0.156
Negative	61 (49.2)	70 (55.1)		25 (73.5)	16 (53.3)	
Positive	63 (50.8)	57 (44.9)		9 (26.5)	14 (46.7)	
**PR**			1.000			0.088
Negative	82 (66.1)	85 (66.9)		28 (82.4)	18 (60.0)	
Positive	42 (33.9)	42 (33.1)		6 (17.6)	12 (40.0)	
**HER2**			0.929			0.575
IHC++/FISH+	23 (18.5)	22 (17.3)		4 (11.8)	6 (20.0)	
IHC+++	101 (81.5)	105 (82.7)		30 (88.2)	24 (80.0)	
**Ki-67 (%)**			0.003*			1.000
≤ 30	68 (54.8)	45 (35.4)		11 (32.4)	9 (30.0)	
> 30	56 (45.2)	82 (64.6)		23 (67.6)	21 (70.0)	
**TILs**			0.069			0.471
High	65 (52.4)	51 (40.2)		20 (58.8)	14 (46.7)	
Low	59 (47.6)	76 (59.8)		14 (41.2)	16 (53.3)	
**TRAG**			0.560			0.431
Wild-type	114 (91.9)	113 (89.0)		30 (88.2)	29 (96.7)	
Mutant	10 (8.1)	14 (11.0)		4 (11.8)	1 (3.3)	

CNA: Copy number alteration; y/o: years old; BCS: breast-conserving surgery; ALN: axillary lymph node; ER: estrogen receptor; PR: progesterone receptor; HER2: human epidermal growth factor receptor 2; TILs: tumor-infiltrating lymphocytes; TRAG: Trastuzumab response-associated gene signature. *Two-sided $P < 0.05$.

Supplementary Table 5. Demographical characteristics of the HER2 positive breast cancer patients according to recurrent status in the TCGA cohort

Characteristics	Total<br>N = 62 (%)	Sensitive<br>N = 56 (%)	Resistant<br>N = 6 (%)	P value
**Age (y/o)**				0. 689
≤ 50	20 (32.3)	19 (33.9)	1 (16.7)	
> 50	42 (67.7)	37 (66.1)	5 (83.3)	
**Breast surgery**				1.000
BCS	30 (48.4)	27 (48.2)	3 (50.0)	
Mastectomy	32 (51.6)	29 (51.8)	3 (50.0)	
**Tumor size (cm)**				0.881
≤ 2.0	14 (22.6)	12 (21.4)	2 (33.3)	
> 2.0	48 (77.4)	44 (78.6)	4 (66.7)	
**ALN status**				1.000
Negative	62 (100.0)	56 (100.0)	6 (100.0)	
Positive	0 (0.0)	0 (0.0)	0 (0.0)	
**ER**				1.000
Negative	62 (100.0)	56 (100.0)	6 (100.0)	
Positive	0 (0.0)	0 (0.0)	0 (0.0)	
**PR**				1.000
Negative	62 (100.0)	56 (100.0)	6 (100.0)	
Positive	0 (0.0)	0 (0.0)	0 (0.0)	
**TRAG status**				0.151
Wild-type	42 (67.7)	40 (71.4)	2 (33.3)	
Mutant	20 (32.3)	16 (28.6)	4 (66.7)	

HER2: Human epidermal growth factor receptor 2; TCGA: the Cancer Genome Atlas; y/o: years old; BCS: breast conserving surgery; ALN: axillary lymph node; ER: estrogen receptor; PR: progesterone receptor; TRAG: trastuzumab response-associated gene signature.

Supplementary Table 6. Baseline characteristics of RJBC cohort according to trastuzumab response-associated gene signature (TRAG) status

	Training (N = 251)			Validation (N = 64)		
**Characteristics**	**Wild-type N = 214 (%)**	**Mutant N = 37 (%)**	**P value**	**Wild-type N = 57 (%)**	**Mutant N =7 (%)**	**P value**
**Age (y/o)**			1.000			0.147
≤ 50	77 (36.0%)	13 (35.1%)		20 (35.1%)	5 (71.4%)	
> 50	137 (64.0%)	24 (64.9%)		37 (64.9%)	2 (28.6%)	
**Breast surgery**			0.239			0.745
BCS	47 (22.0%)	12 (32.4%)		16 (28.1%)	1 (14.3%)	
Mastectomy	167 (78.0%)	25 (67.6%)		41 (71.9%)	6 (85.7%)	
**Tumor size (cm)**			0.100			0.125
≤ 2.0	92 (43. 0%)	10 (27.0%)		21 (36.8%)	0 (0.0%)	
> 2.0	122 (57.0%)	27 (73.0%)		36 (63.2%)	7 (100.0%)	
**ALN status**			0.297			0.713
Negative	104 (48.6%)	22 (59.5%)		32 (56.1%)	5 (71.4%)	
Positive	110 (51.4%)	15 (40.5%)		25 (43.9%)	2 (28.6%)	
**Grade**			0.876			0.352
I-II	69 (32.2%)	13 (35.1%)		23 (40.4%)	1 (14.3%)	
III	145 (67.8%)	24 (64.9%)		34 (59.6%)	6 (85.7%)	
**ER**			0.435			0. 397
Negative	109 (50.9%)	22 (59.5%)		35 (61.4%)	6 (85.7%)	
Positive	105 (49.1%)	15 (40.5%)		22 (38.6%)	1 (14.3%)	
**PR**			0.277			0.676
Negative	139 (65.0%)	28 (75.7%)		40 (70.2%)	6 (85.7%)	
Positive	75 (35.0%)	9 (24.3%)		17 (29.8%)	1 (14.3%)	
**HER2**			1.000			0.654
IHC++/FISH+	38 (17.8%)	7 (18.9%)		8 (14.0%)	2 (28.6%)	
IHC+++	176 (82.2%)	30 (81.1%)		49 (86.0%)	5 (71.4%)	
**Ki-67 (%)**			0.763			0.552
≤ 30	95 (44.4%)	18 (48.6%)		19 (33.3%)	1 (14.3%)	
> 30	119 (55.6%)	19 (51.4%)		38 (66.7%)	6 (85.7%)	
**TILs**			0.830			0.531
High	100 (46.7%)	16 (43.2%)		29 (50.9%)	5 (71.4%)	
Low	114 (53.3%)	21 (56.8%)		28 (49.1%)	2 (28.6%)	
**CNA**			1.000			0.531
Low	106 (49.5%)	18 (48.6%)		29 (50.9%)	5 (71.4%)	
High	108 (50.5%)	19 (51.4%)		28 (49.1%)	2 (28.6%)	

RJBC: Ruijin Hospital Comprehensive Breast Health Center; TRAG: trastuzumab response-associated gene signature; y/o: years old; BCS: breast conserving surgery; ALN: axillary lymph node; ER: estrogen receptor; PR: progesterone receptor; HER2: human epidermal growth factor receptor 2; TILs: tumor-infiltrating lymphocytes; CNA: copy number alteration.

Supplementary Table 7. Baseline characteristics of RJBC cohort according to TILs level

	Training (N = 251)			Validation (N = 64)		
**Characteristics**	**High-level N = 116 (%)**	**Low-level N = 135 (%)**	**P value**	**High-level N = 34 (%)**	**Low-level N = 30 (%)**	**P value**
**Age (y/o)**			0.615			1.000
≤ 50	44 (37.9)	46 (34.1)		13 (38.2)	12 (40.0)	
> 50	72 (62.1)	89 (65.9)		21 (61.8)	18 (60.0)	
**Breast surgery**			0.014*			0.049*
BCS	36 (31.0)	23 (17.0)		13 (38.2)	4 (13.3)	
Mastectomy	80 (69.0)	112 (83.0)		21 (61.8)	26 (86.7)	
**Tumor size (cm)**			0.869			1.000
≤ 2.0	46 (39.7)	56 (41.5)		11 (32.4)	10 (33.3)	
> 2.0	70 (60.3)	79 (58.5)		23 (67.6)	20 (66.7)	
**ALN status**			0.748			0.051
Negative	60 (51.7)	66 (48.9)		24 (70.6)	13 (43.3)	
Positive	56 (48.3)	69 (51.1)		10 (29.4)	17 (56.7)	
**Grade**			0.235			0.518
I-II	33 (28.4)	49 (36.3)		11 (32.4)	13 (43.3)	
III	83 (71.6)	86 (63.7)		23 (67.6)	17 (56.7)	
**ER**			0.992			0.370
Negative	60 (51.7)	71 (52.6)		24 (70.6)	17 (56.7)	
Positive	56 (48.3)	64 (47.4)		10 (29.4)	13 (43.3)	
**PR**			0.855			0.251
Negative	76 (65.5)	91 (67.4)		27 (79.4)	19 (63.3)	
Positive	40 (34.5)	44 (32.6)		7 (20.6)	11 (36.7)	
**HER2**			0.816			0.897
IHC++/FISH+	22 (19.0)	23 (17.0)		6 (17.6)	4 (13.3)	
IHC+++	94 (81.0)	112 (83.0)		28 (82.4)	26 (86.7)	
**Ki-67 (%)**			<0.001**			0.251
≤ 30	38 (32.8)	75 (55.6)		8 (23.5)	12 (40.0)	
> 30	78 (67.2)	60 (44.4)		26 (76.5)	18 (60.0)	
**CNA**			0.069			0.471
Low	65 (56.0)	59 (43.7)		20 (58.8)	14 (46.7)	
High	51 (44.0)	76 (56.3)		14 (41.2)	16 (53.3)	
**TRAG**			0.011*			1.000
Wide-type	103 (88.8)	102 (75.6)		29 (85.3)	25 (83.3)	
Mutant	13 (11.2)	33 (24.4)		5 (14.7)	5 (16.7)	

RJBC: Ruijin Hospital Comprehensive Breast Health Center; TILs: tumor-infiltrating lymphocytes; y/o: years old; BCS: breast-conserving surgery; ALN: axillary lymph node; ER: estrogen receptor; PR: progesterone receptor; HER2: human epidermal growth factor receptor 2; CNA: copy number alteration; TRAG: trastuzumab response-associated gene signature. *Two-sided $P < 0.05$, **Two-sided $P < 0.001$.

Supplementary Table 8. Univariate and multivariate analysis of clinicopathological features, TILs and TRAG status in relation to disease-free survival in the training cohort

Variables	Univariate analysis		Multivariate analysis	
	HR (95%CI)	P value	HR (95%CI)	P value
Age (≤50 y/o *vs.* >50 y/o)	1.22 (0.75 – 1.99)	0.417	/	/
Breast surgery (Mastectomy *vs.* BCS)	1.54 (0.83 – 2.88)	0.174	/	/
Tumor size (>2.0cm *vs.* ≤2.0cm)	3.45 (1.74 – 6.85)	< 0.001**	3.24 (1.62 – 6.45)	0.001*
ALN status (positive *vs.* negative)	1.79 (1.05 – 3.08)	0.034*	2.05 (1.17 – 3.58)	0.012*
Tumor grade (III *vs.* I-II)	1.50 (0.92 – 2.43)	0.104	/	/
ER (negative *vs.* positive)	3.15 (1.69 – 5.86)	< 0.001**	3.13 (1.67 – 5.87)	<0.001**
HER2 (+++ *vs.* ++/FISH+)	0.95 (0.51 – 1.77)	0.866	/	/
Ki-67 (>30% *vs.* ≤30%)	0.80 (0.49 – 1.29)	0.355	/	/
TILs (≤15% *vs.* >15%)	2.43 (1.36 - 4.35)	0.003*	2.65 (1.47 – 4.76)	0.001*
CNA (high *vs.* low)	1.51 (0.87 – 2.61)	0.140	/	
TRAG mutation (yes *vs.* no)	3.44 (1.78 – 6.67)	< 0.001**	3.51 (1.77 – 6.96)	< 0.001**

TILs: Tumor-infiltrating lymphocytes; TRAG: trastuzumab response-associated gene signature; HR: hazard Ratio; CI: confidence interval; y/o: years old; BCS: breast-conserving surgery; ALN: axillary lymph node; ER: estrogen receptor; PR: progesterone receptor; HER2: human epidermal growth factor receptor 2; CNA: copy number alteration. *Two-sided $P < 0.5$. **Two-sided $P < 0.001$.
